# A content validity and cognitive interview process to evaluate an Illness Perception Questionnaire for African Americans with type 2 diabetes

**DOI:** 10.1186/s13104-019-4342-9

**Published:** 2019-05-30

**Authors:** Olayinka O. Shiyanbola, Daniel Bolt, Adati Tarfa, Carolyn Brown, Earlise Ward

**Affiliations:** 1 Division of Social and Administrative Sciences School of Pharmacy, University of Wisconsin-Madison, 777 Highland Avenue, Madison, WI 53705 USA; 2 Department of Educational Psychology, University of Wisconsin-Madison, Madison, WI USA; 3 Division of Health Outcomes and Pharmacy Practice, University of Texas-Austin, Austin, TX USA; 4 Department of Nursing, University of Wisconsin-Madison, Madison, WI USA

**Keywords:** African Americans, Illness perception, Cognitive interviews, Diabetes

## Abstract

**Objective:**

The Illness Perception Questionnaire (IPQ) is the only available and empirically validated tool used to gain insight into patient illness beliefs. However, the IPQ has reliability and validity problems when used with African Americans (AAs) and needs to be culturally-adapted and validated for use with this group. This study aimed to utilize findings from focus groups to culturally adapt the IPQ for use in AAs with diabetes. Ten cognitive interviews among AAs with type 2 diabetes explored patients’ interpretation and understanding of the adapted IPQ.

**Results:**

Forty-four new survey items were added to the IPQ. Twenty-nine of the forty-four items were determined as the appropriate number of questions to be tested because of time, and to reduce respondent burden. After the first round of interviews, an item-by-item review of the new items identified problems related to AA comprehension of certain items, their applicability, and wording/tone. Five items identified as problematic were related to AAs understanding of a cure for diabetes, their perception of how food influences their diabetes, how their identity as AAs influence diabetes control, and the dialogue about diabetes within their families and/or community. Findings support the newly developed illness perception questions as culturally specific to AAs with diabetes after being tested for content validity and participant understanding using cognitive interviews.

**Electronic supplementary material:**

The online version of this article (10.1186/s13104-019-4342-9) contains supplementary material, which is available to authorized users.

## Introduction

Type 2 diabetes, a highly burdensome chronic illness affects 3.7 African Americans (AAs) in the United States and leads to a greater risk of death and disability compared to non-Hispanic whites [1]. AAs are also likely to develop diabetes-related complications such as heart disease, stroke, and amputations that eventually lead to death, compared to non-Hispanic whites [2]. A prominent contributor to these diabetes health disparities is poor medication adherence [3]. Medication adherence is defined by the World Health Organization as the extent to which patients take their medication in correspondence with the agreed recommendations from a health care provider [4]. Poor medication adherence causes 50% of treatment failures, leading to 125,000 deaths and 69% of medication-related admissions [2, 5–8]. While there are many reasons for poor medication adherence, compared to patient sociodemographic and clinical factors, patient beliefs about their illness has one of the largest influences on medication adherence [9, 10].

Poor medication adherence among AAs may be partly due to the lack of personalized and culturally-adapted interventions that account for their unique illness beliefs which may be different compared to other racial groups [3, 11]. Also, the differences in diabetes complications between AAs and whites may result from these overlooked illness beliefs. For example, compared to whites, some AAs have religious beliefs about the control of diabetes including a belief in God’s ability to cure diabetes [11]. One of the important steps in improving medication adherence among AAs with diabetes is to assess their beliefs about diabetes in a valid and reliable way. The only validated and reliable measure available to assess illness beliefs is the Illness Perception Questionnaire (IPQ). The components of the survey provide a framework for patients to make sense of their symptoms, assess their health risk, and direct their actions and coping skills. However, this survey has only been validated in a Western European population [12, 13] and has significant reliability and validity measurement problems when used with AAs [14–17]. For example, in a study by Ward et al. [15], five of the revised Illness Perception Questionnaire sub-scales had consistency/accuracy issues when used among AAs with mental illness in relation to their health-seeking behavior [16]. The measure did not address unique AA cultural beliefs that defined their coherent view of their mental illness. The challenges in measuring beliefs about mental illness might be similar when measuring beliefs about diabetes. For example, AA diabetes-related cultural beliefs such as familial relationships, their racial identity and/or religious beliefs are not accounted for in the IPQ. Thus, there is an urgent need for a valid and reliable culturally-adapted survey to assess illness beliefs for AAs. Importantly, to develop and implement culturally appropriate diabetes medication adherence interventions which account for AAs illness beliefs, a measure that appropriately assesses their culturally informed beliefs is needed.

In evaluating and making changes to a survey tool, it is important to understand if added or revised items measure what the researchers intend and respondents understand and correctly interpret the items [18]. For this study, subsequent to the adaptation of the Illness Perception Questionnaire for AAs with diabetes, it was important to ensure the adapted questionnaire had content (survey items) that represented illness perceptions and was understandable to AAs. Hence, cognitive interviewing (a psychologically oriented method for empirically studying how individuals mentally process and respond to survey questionnaires) [19] was used as a means to pre-test the culturally adapted IPQ to explore if the new survey items measured what was intended and whether respondents understood and could respond to the items.

### Objective

The study aimed to utilize qualitative findings from prior focus groups [20, 21] to adapt the established Illness Perception Questionnaire [13, 22] for use in AAs with diabetes, and to use cognitive interviews to explore patients’ interpretation and understanding of the survey. This study describes the cognitive interviewing process used to pretest the survey. The cognitive interviews were an important pre-survey evaluation step in the questionnaire development process because it identifies response errors, and can inform early identification of questionnaire problems [23]. We discuss the methods that went into refining and validating the questionnaire in this early development process. Cognitive interviewing identifies potential response problems that may compromise the response rate and the quality of the data and are used in creating culturally appropriate questionnaires for diverse populations. The items selected for evaluation were significantly different in context and topic compared to the original items.

## Main text

### Methods

#### Study design

Given the focus of this study, a qualitative approach with the use of cognitive interviewing seemed the best fit. Cognitive interviewing identifies sources of response error in survey questionnaires by focusing on the cognitive process respondents use to answer questions [18, 19, 24, 25].

To prepare for the cognitive interviews, we conducted the following:

1. Based on the revised IPQ measure [22], an adapted survey with culturally-embedded characterizations of illness beliefs was developed using themes generated from six focus groups of 40 AAs with diabetes [20, 21].
2. Forty-four new items were added to the 39-item revised IPQ, resulting in a culturally adapted tool with 83 survey items (Additional file 1: Appendix).

Two experts on illness perceptions (EW and CB), as well as an expert on survey design and psychometric methods (DB), examined the survey for content areas needed to measure the illness beliefs important to AAs medication adherence, as well as survey item structure and wording. Then, the cognitive interviews were completed.

#### Sample

Six women and four men English-speaking AA age 45–60 years were recruited using purposive sampling. The inclusion criteria were self-identified AA/Blacks who spoke English, self-reported diagnosis of type 2 diabetes by a health care provider at least 1 year prior to the time of the study and reported taking at least one diabetes medication by mouth. The cognitive interviews were conducted 2 weeks apart in March 2018. The study was approved by the University of Wisconsin-Madison Health Sciences Institutional Review Board (IRB number: 2018-0281).

#### Recruitment

Study participants were recruited from different AA community locations in a Midwestern City in the United States. Flyers were distributed at AA churches, non-profit organizations, public libraries, and community centers. Interested participants then contacted the study recruiter about their intention to participate. Informed consent was obtained prior to the interviews.

#### Measures and data collection procedures

##### Interviews

Interviews were conducted in private locations at the convenience of participants. Two iterative interview processes were conducted 2 weeks apart, with 5 respondents in each phase. All interviews were audio-recorded. The survey was administered by an AA Ph.D. trained health-equity researcher and an AA project assistant trained in facilitating cognitive interviews. At the start of the interview, standardized instructions were read aloud to each participant. Respondents were told that the essence of the interview was not to test respondents for right or wrong answers. Rather, it was to understand their thought process and understanding of questions and to explore any suggestions or comments they had about the questions.

##### Probing methods

Verbal probing (a form of data collection in which the cognitive interviewer administers a series of probe questions specifically designed to elicit detailed information beyond that normally provided by respondents) was used [26]. The interviewers guided the interaction more proactively by asking additional, direct questions about the basis of respondents’ responses. Respondents were offered a copy of the Likert-scale and asked to identify their response choice following each question by the interviewer. Sample questions included, “*There is no cure for your diabetes. Do you strongly agree, agree, neither agree nor disagree, disagree, or strongly disagree?*” The interviewer further probed the respondents of their answer choices. Probing questions were tailored to the respondents’ replies. For example, *“Tell me more about why you answered [ANSWER] for this question.”* If a participant chose “neither agree nor disagree” they were further probed to decide if they had to choose between “agree” or “disagree,” which option would they have selected and the reason for that choice. Salient words in the questions were selected and further probed. For example, the interviewer asked, “*What did the word “cure” mean to you in this question?”* For each question, respondents were encouraged to share any suggestions or concerns regarding the questions. To ensure that respondents only focused on each separate survey question per time, only the pertinent question was visible to the respondents at each time.

Think aloud methods (which requests that the survey respondents actively verbalize their thoughts as they attempt to answer the survey questions) were also used as a cognitive process for exploring participants’ understanding [27]. The respondents used this think-aloud technique to talk through the process of their answer selection. Basic demographic characteristics were collected prior to the interviews. Participants were given $50 cash upon completion of the interviews.

#### Data analysis

All interviews were transcribed by a certified transcriptionist. Thematic analysis was used to analyze the interviews by combining the notes pertaining to each evaluated survey item, exploring and aggregating common themes across interviews, and identifying key findings that may indicate differences between the intended interpretation and that of respondents [28]. The analysis was completed by two individuals trained in qualitative data analysis. Data analysis was conducted using Nvivo 11 (QSR International-Melbourne) and continued until the researchers could not identify newer dimensions within the data (data saturation). After all analyses were completed independently, the coders met to identify similarities and differences between themes and discuss any divergences. Data analysis was completed upon data saturation.

### Results

Ten AA men and women participated. Most respondents were female (n = 6, 60.0%) with a mean age of 57.5 (± 4.5) years. Each participant evaluated each survey item and response option presented to them. The experts on illness perceptions, survey design, and psychometric methods determined that each new survey item was important in the measurement of AA beliefs about diabetes. Using face value, they evaluated each new item for how important it was in the conceptualization of AAs’ illness beliefs. If the survey item did not seem to represent AAs beliefs, they indicated this in their evaluation. Hence, no items were deleted.

#### Cognitive interviews

After the interviews, an item-by-item review of the 29 new survey items identified problems with five items related to AA’s comprehension, reference/applicability, and wording/tone. These items were related to AA’s understanding of a cure for diabetes, perception of how food influences their diabetes, how their identity as AA influences diabetes control, and the dialogue about diabetes within their families and/or community (Table 1). For the problem types, *comprehension of question* was related to their understanding of what the question meant, *unclear reference* focused on their ability to connect the question to their life and answer appropriately, *wording or tone* referred to their sensitivity to the language/word used. Five of the items were revised to make them clearer and no questions were deleted (Table 2).

**Table 1:** Questionnaire items tested in cognitive interviews and found to be confusing or misinterpreted

Question tested	Purpose of the question	Sample respondents’ quotes	Problem type identified
There is no cure for my diabetes	To understand if AAs believe that there is a cure for diabetes and the cure lies in exercise, weight loss and eating health	*“I agree. Because I’ve been told by the dietician, by the doctor, by mostly everybody you talk to in the health field that that is the way to mostly have it to disappear is by eating the right foods, exercise play a most important part in it. And so it’s, like again, it’s up to you to do those things* *Mostly everybody you talk to in the health field that that is the way to mostly have it to disappear is by eating the right foods, exercise play a most important part in it. And so it’s, like again, it’s up to you to do those things.”*—*002*	Comprehension of Question
My diabetes has taken away my ability to enjoy the food I grew up eating	To understand the impact of diabetes on the food AAs grew up eating and enjoying	*“Yes, I am. Ooh, I have to watch now what I pretty much the greasiness and all the food we used to eat growing up, ham hocks and different things like that, the way you cook them. You got to re*-*do it now, which mean you got to keep out some of that butter and lard and grease and all that kind of stuff. So you got to cook a different way. This is only one, you know. But anyway, it’s good for you, to make a change.”*—*003* *“Yeah, I’m back with the nutritionist now, but I haven’t seen, the lady I was seeing, she retired. And like I said, I went to meet my new nutritionist, and I got my log book, and you just remember, I meant to put my pedometer on to do my steps. So it’s important to me, I think, to keep a food diary.”*—*002*	Comprehension of Question
Because of my physical and mental health, it is important to not worry about my diabetes	To understand if AA try not to worry about their diabetes to preserve their mental health and physical health	*“Because medications, and which I was already borderline, but the medications that I was taking, steroids, and psychotropics, like blew me up. Car accidents, back on steroids* *Depression, back on the psych meds. So a lot of them had kind of led to the diabetes. So now the exercising, I’m trying to get back down to my normal size. I’m still working on it. I’m down two sizes now. And I’m trying to get this off.”*—*007* *“It means that because where I am physically and mentally, it, I need not to worry about diabetes. Right, so basically, I’m hearing it say that because of where I’m at physically and mentally, it’s important not, for me not to be worrying about my, you know, diabetes in my life.”*—*004*	Unclear Reference
As a person of my racial identity, I have to advocate for myself if I want to survive with diabetes	To examine if AA’s feel an added pressure to advocate for diabetes care due to their long history of distrust in healthcare professionals	*“Well, you know, I’ve taken some pills that, you know, that they’ve given me that didn’t do anything. And once, you know, I’ll take it for a period of time, and if it’s not doing anything I stop it, you know, I go noncompliant.”*—*004*	Comprehension of question
Diabetes is a silent disease not discussed within my community	To examine if there is stigma associated with diabetes in AA communities or it is an illness openly discussed in their communities	*“When you think of silent, there’s a couple ways you can possibly look at it. And like I said, one way is a disease that silently creeps up on you. Or the other way is a silent disease meaning that people don’t have discussions or don’t really talk about it in, within my community.”*—*009* *“When I think like, let me see how it is in context. I guess, hush*–*hush, like people don’t discuss it or have dialogue about it. The other part that I take from that silent disease is like it’s almost like a disease is rare or, you know, that it just sneak up on you violently. So I take it both ways.”*—*005*	Wording or tone

**Table 2:** Changes made to new survey questions based on participant feedback

Problematic questions	Changes made to the questions
Diabetes is a silent disease not discussed within my community	Diabetes is a disease not discussed within the black community
My diabetes has taken away my ability to enjoy the food I grew up eating	My diabetes has taken away my ability to eat the food I grew up eating
There is no cure for my diabetes	There is a known cure for diabetes
Because of my physical and mental health, it is important to not worry about my diabetes	It is important not to worry about my diabetes so as to protect my physical and mental health
As a person of my racial identity, I have to advocate for myself if I want to survive with diabetes	As a black person, I have to advocate for myself if I want to live with diabetes

### Discussion

Our findings suggest that to create a culturally adapted survey that is usable by AAs, focusing on the target population perception of the meaning of the questions prior to survey administration is fundamental in the design of the survey. Listening to and probing respondents as they verbalized their thought processes allowed for the refinement of the survey. Out of 29 newly developed survey items that were culturally specific to AAs with diabetes and tested using cognitive interviews, five questions were identified as problematic and revised accordingly.

The study results show that by utilizing cognitive interviewing, the comprehension of questions for a culturally adapted illness perception questionnaire can be improved and made culturally appropriate for underserved populations, and questions can be easily interpreted when they are more applicable to the daily life of respondents. By better addressing respondents’ concern about the new survey items in the culturally adapted IPQ, more reliable and valid data on AAs illness beliefs about type 2 diabetes can be collected and utilized for clinical and research purposes. For example, the adapted questionnaire can be offered to AA patients with poor control of diabetes and/or medication nonadherence to understand which illness beliefs should be addressed in an intervention.

Although the Illness Perception Questionnaire has been pretested in different populations, no intensive cognitive interviewing process had been conducted before prior use in an AA population. This study’s results further support that cognitive interviews are an effective technique for improving health surveys and modifying them to be culturally relevant to diverse populations [19, 26]. The finalized version of the culturally adapted Illness Perception Questionnaire for AAs with diabetes will be further tested for reliability and validity among a larger group of AAs with type 2 diabetes.

### Conclusion

The study findings support the newly developed illness perception questions as culturally specific to AAs with diabetes after being tested for content validity and participants’ understanding using cognitive interviews.

## Limitations

The study had some limitations. General probes were administered before specific probes for most questions. It is possible that the response to the specific probes was enhanced because participants had been primed from the general probes. The respondents were selected from a single city in a Midwestern, United States town. AAs from more diverse locations may have a different interpretation of the survey questions. Future studies should conduct further cognitive testing among AAs from different parts of the United States. Due to the time constraints of the cognitive interview process, only 29 survey items out of the 44 new items were tested.

## Additional file

**Additional file 1: Appendix.** A preliminary version of the Culturally Adapted Illness Perception Questionnaire for African Americans with Diabetes. This is the preliminary version of the Culturally Adapted Illness Perception Questionnaire for African Americans with Diabetes.

## Data Availability

The dataset used and/or analyzed for the current study are available from the corresponding author on reasonable request.

## Abbreviation

AAs: African Americans
