# Supplementary material for: A content validity and cognitive interview process to evaluate an Illness Perception Questionnaire for African Americans with type 2 diabetes
Source: BMC Res Notes. 2019 May 30;12:308. doi: 10.1186/s13104-019-4342-9 (PMC6543586; doi:10.1186/s13104-019-4342-9)
Supplement: Supplementary file 1 — Additional file 1: Appendix. A preliminary version of the Culturally Adapted Illness Perception Questionnaire for African Americans with Diabetes. This is the preliminary version of the Culturally Adapted Illness Perception Questionnaire for African Americans with Diabetes. [file 13104_2019_4342_MOESM1_ESM.docx]

Appendix

A preliminary version of the Culturally Adapted Illness Perception Questionnaire for African Americans with Diabetes

**YOUR VIEWS ABOUT YOUR DIABETES**

**Listed below are a number of symptoms that you may or may not have experienced since your diabetes. Please indicate by circling *Yes* or *No*, whether you have experienced any of these symptoms since your diabetes, and whether you believe that these symptoms are related to your diabetes.**

**I have experienced this symptom *since my diabetes***

| **Pain** | **Yes** | **No** |  | **Yes** | **No** |
| --- | --- | --- | --- | --- | --- |
| **Sore Throat** | **Yes** | **No** |  | **Yes** | **No** |
| **Nausea** | **Yes** | **No** |  | **Yes** | **No** |
| **Breathlessness** | **Yes** | **No** |  | **Yes** | **No** |
| **Weight Loss** | **Yes** | **No** |  | **Yes** | **No** |
| **Fatigue** | **Yes** | **No** |  | **Yes** | **No** |
| **Stiff Joints** | **Yes** | **No** |  | **Yes** | **No** |
| **Sore Eyes** | **Yes** | **No** |  | **Yes** | **No** |
| **Wheeziness** | **Yes** | **No** |  | **Yes** | **No** |
| **Headaches** | **Yes** | **No** |  | **Yes** | **No** |
| **Upset Stomach** | **Yes** | **No** |  | **Yes** | **No** |
| **Sleep Difficulties** | **Yes** | **No** |  | **Yes** | **No** |
| **Dizziness** | **Yes** | **No** |  | **Yes** | **No** |
| **Loss of Strength** | **Yes** | **No** |  | **Yes** | **No** |

**This symptom is *related to my diabetes***

We are interested in your own personal views of how you see your current diabetes. Please indicate how much you agree or disagree with the following statements about your diabetes by checking the appropriate box. **(N- New item for sub-scale, O- Old item for sub-scale)**

|  |  | Strongly disagree | disagree | neither agree nor disagree | agree | stRongly agree |
| --- | --- | --- | --- | --- | --- | --- |
| TMn | Diabetes can be reversed |  |  |  |  |  |
| Tmn | Nothing can make my diabetes go away |  |  |  |  |  |
| 1. TMN | There is a known cure for diabetes |  |  |  |  |  |
| 1. TMn | My diabetes could go away if I exercise, lose weight and eat healthy |  |  |  |  |  |
| 1. TMo | My diabetes will last a short time |  |  |  |  |  |
| 1. TMo | My diabetes is likely to be permanent rather than temporary |  |  |  |  |  |
| 1. TMo | My diabetes will last for a long time |  |  |  |  |  |
| 1. TMo | I expect to have this diabetes for the rest of my life |  |  |  |  |  |
| 1. CON | My diabetes reduces the control I have over my life |  |  |  |  |  |
| 1. COn | My diabetes has harmed my relationship with others close to me |  |  |  |  |  |
| 1. CON | My diabetes has caused difficulties in my relationships with family and friends |  |  |  |  |  |
| CON | My diabetes has caused my relationships with family and friends to be less close |  |  |  |  |  |
| CON | My diabetes reduces my participation in social activities within the community |  |  |  |  |  |
| CON | My diabetes takes away the ability to enjoy food in my daily life |  |  |  |  |  |
| CON | Having diabetes has kept me away from the type of job I want to have |  |  |  |  |  |
| CON | My diabetes has taken away my ability to eat the food I grew up eating |  |  |  |  |  |
| COO | My diabetes is a serious condition |  |  |  |  |  |
| COO | My diabetes has major consequences on my life |  |  |  |  |  |
| COO | My diabetes strongly affects the way others see me |  |  |  |  |  |
| PCN | My diabetes is a big part of who I am |  |  |  |  |  |
| PCN | Having diabetes has made me feel less like a strong, black, person |  |  |  |  |  |
| PCN | It is important not to worry about my diabetes so as to protect my physical and mental health |  |  |  |  |  |
| PCN | Faith in God helps control my diabetes |  |  |  |  |  |
| PCN | God helps me not to worry about my diabetes |  |  |  |  |  |
| PCN | My friends and family encourage me to manage my diabetes |  |  |  |  |  |
| PCO | I have the power to influence my diabetes |  |  |  |  |  |
| PCO | Nothing I do will affect my diabetes |  |  |  |  |  |
| PCO | My actions will have no affect on the outcome of my diabetes |  |  |  |  |  |
| TCN | Medications can help with my diabetes |  |  |  |  |  |
| TCN | Medications can help me survive with my diabetes |  |  |  |  |  |
| TCO | There is very little that can be done to improve my diabetes |  |  |  |  |  |
| TCO | My treatment will be effective in curing my diabetes |  |  |  |  |  |
| TCO | The negative effects of my diabetes can be prevented (avoided) by my treatment |  |  |  |  |  |
| TCO | My treatment can control my diabetes |  |  |  |  |  |
| TCO | There is nothing which can help my condition |  |  |  |  |  |
| ICN | How I got diabetes is a mystery to me |  |  |  |  |  |
| ICN | I understand how I got diabetes |  |  |  |  |  |
| ICO | The symptoms of my condition are puzzling to me |  |  |  |  |  |
| ICO | My diabetes doesn’t make any sense to me |  |  |  |  |  |
| ICO | I have a clear picture or understanding of my condition |  |  |  |  |  |
| TCO | The symptoms of my diabetes change a great deal from day to day |  |  |  |  |  |
| TLC-O | My symptoms come and go in cycles |  |  |  |  |  |
| TLC-o | My diabetes is very unpredictable |  |  |  |  |  |
| TLC-O | I go through cycles in which my diabetes gets better and worse |  |  |  |  |  |
| ERN | I am scared of having complications from my diabetes |  |  |  |  |  |
| ERN | The experiences of my family and friends has led me to fear diabetes complications |  |  |  |  |  |
| ERN | Having diabetes makes me worry about my future |  |  |  |  |  |
| ERN | I am worried my diabetes will stop me from seeing my children and grandchildren grow up |  |  |  |  |  |
| ERN | It is hard for me to accept that I have diabetes |  |  |  |  |  |
| ERN | It makes me mad that I have to change my life because of diabetes |  |  |  |  |  |
| ERN | I am frustrated with having diabetes |  |  |  |  |  |
| ERN | I am depressed because I have diabetes |  |  |  |  |  |
| ERN | My diabetes controls my life |  |  |  |  |  |
| ERN | I am upset I have diabetes |  |  |  |  |  |
| ERN | I refuse to be depressed because I have diabetes |  |  |  |  |  |
| ERN | I am concerned about dying from my diabetes |  |  |  |  |  |
| ERN | I am worried about my children/grandchildren getting diabetes |  |  |  |  |  |
| ERO | I get depressed when I think about my diabetes |  |  |  |  |  |
| ERO | When I think about my diabetes I get upset |  |  |  |  |  |
| ERO | My diabetes makes me feel angry |  |  |  |  |  |
| ERO | My diabetes does not worry me |  |  |  |  |  |
| ERO | Having this diabetes makes me feel anxious |  |  |  |  |  |
| ERO | My diabetes makes me feel afraid |  |  |  |  |  |
| SD | As a Black person, I have to advocate for myself if you want to live with diabetes |  |  |  |  |  |
| SD | Being Black decreases my chances of knowing about diabetes control |  |  |  |  |  |
| SD | Being Black reduces my chances of getting information about diabetes |  |  |  |  |  |
| SD | Being Black makes me more likely to get diabetes |  |  |  |  |  |
| SD | Diabetes is a disease not discussed within the Black community |  |  |  |  |  |
| SD | My friends and family discuss diabetes |  |  |  |  |  |
| SD | My friends and family discourage me from being open about my diabetes |  |  |  |  |  |
| SD | My friends and family help me learn about my diabetes |  |  |  |  |  |
| SD | Being poor influences whether I get diabetes |  |  |  |  |  |

**CAUSES OF MY DIABETES**

**We are interested in what you consider may have been the cause of your diabetes. As people are very different, there is no correct answer for this question. We are most interested in your own views about the factors that caused your diabetes rather than what others including doctors or family may have suggested to you. Below is a list of possible causes for your diabetes. Please indicate how much you agree or disagree that they were causes for you by ticking the appropriate box.**

|  | **POSSIBLE CAUSES** | **STRONGLY DISAGREE** | **DISAGREE** | **NEITHER AGREE NOR DISAGREE** | **AGREE** | **STRONGLY AGREE** |
| --- | --- | --- | --- | --- | --- | --- |
| **CO** | **Stress or worry** |  |  |  |  |  |
| **CO** | **Hereditary - it runs in my family** |  |  |  |  |  |
| **CO** | **A Germ or virus** |  |  |  |  |  |
| **CO** | **Diet or eating habits** |  |  |  |  |  |
| **CO** | **Chance or bad luck** |  |  |  |  |  |
| **CO** | **Poor medical care in my past** |  |  |  |  |  |
| **CO** | **Pollution in the environment** |  |  |  |  |  |
| **CO** | **My own behavior** |  |  |  |  |  |
| **CO** | **My mental attitude e.g. thinking about life negatively** |  |  |  |  |  |
| **CO** | **Family problems or worries** |  |  |  |  |  |
| **CO** | **Overwork** |  |  |  |  |  |
| **CO** | **My emotional state e.g. feeling down, lonely, anxious, empty** |  |  |  |  |  |
| **CO** | **Ageing** |  |  |  |  |  |
| **CO** | **Alcohol** |  |  |  |  |  |
| **CO** | **Smoking** |  |  |  |  |  |
| **CO** | **Accident or injury** |  |  |  |  |  |
| **CO** | **My personality** |  |  |  |  |  |
| **CO** | **Altered immunity** |  |  |  |  |  |
| **CN** | **Medicines** |  |  |  |  |  |
| **CN** | **Cultural foods** |  |  |  |  |  |
| **CN** | **Curses and Ancestors** |  |  |  |  |  |
| **CN** | **Chemicals in food and vaccines** |  |  |  |  |  |
| **CN** | **God’s punishment** |  |  |  |  |  |
| **CN** | **Lack of exercise** |  |  |  |  |  |
| **CN** | **Government conspiracy** |  |  |  |  |  |

**In the table below, please list in rank-order the three most important factors that you now believe caused YOUR diabetes. You may use any of the items from the box above, or you may have additional ideas of your own.**

**The most important causes for me:**

1.

2.

3.
